# Development, characterization and comparisons of targeted and non-targeted metabolomics methods

**DOI:** 10.1371/journal.pone.0207082

**Published:** 2018-11-15

**Authors:** Anton Ribbenstedt, Haizea Ziarrusta, Jonathan P. Benskin

**Affiliations:** 1 Department of Environmental Science and Analytical Chemistry (ACES), Stockholm University, Stockholm, Sweden; 2 Department of Analytical Chemistry, University of the Basque Country (UPV/EHU), Leioa, Basque Country, Spain; University of Pittsburgh, UNITED STATES

## Abstract

The potential of a metabolomics method to detect statistically significant perturbations in the metabolome of an organism is enhanced by excellent analytical precision, unequivocal identification, and broad metabolomic coverage. While the former two metrics are usually associated with targeted metabolomics and the latter with non-targeted metabolomics, a systematic comparison of the performance of both approaches has not yet been carried out. The present work reports on the development and performance evaluation of separate targeted and non-targeted metabolomics methods. The targeted approach facilitated determination of 181 metabolites (quantitative analysis of 18 amino acids, 11 biogenic amines, 5 neurotransmitters, 5 nucleobases and semi-quantitative analysis of 50 carnitines, 83 phosphatidylcholines, and 9 sphingomyelins) using ultra-performance liquid chromatography-tandem mass spectrometry (UPLC-MS/MS) and flow injection-tandem mass spectrometry (FI-MS/MS). Method accuracy and/or precision were assessed using replicate samples of NIST SRM1950 as well as fish liver and brain tissue from Gilthead Bream (*Sparus aurata*). The non-target approach involved UPLC-high resolution (Orbitrap) mass spectrometry (UPLC-HRMS). Testing of ionization mode and stationary phase revealed that a combination of positive electrospray ionization and HILIC chromatography produced the largest number of chromatographic features during non-target analysis. Furthermore, an evaluation of 4 different sequence drift correction algorithms, and combinations thereof, revealed that batchCorr produced the best precision in almost every test. However, even following correction of non-target data for signal drift, the precision of targeted data was better, confirming our existing assumptions about the strengths of targeted metabolomics. Finally, the accuracy of the online MS^2^-library mzCloud was evaluated using reference standards for 38 different metabolites. This is among the few studies that have systematically evaluated the performance of targeted and non-targeted metabolomics and provides new insight into the advantages and disadvantages of each approach.

## Introduction

Metabolomics has become an increasingly powerful research tool in the natural and life sciences for elucidating biological perturbations in response to internal or external stimuli. Strategies for performing metabolomics experiments are classified as either ‘targeted’ or ‘non-targeted’. Targeted approaches involve multiplexed analysis of known metabolites and have proven useful for assessing organismal response with respect to development[1,2] environmental perturbation[1,3,4], xenobiotic exposure[5–7] or disease pathology and diagnosis in the life sciences[8–10]. In comparison, non-targeted approaches seek to detect as many distinct features as possible in a single analysis and, combined with multivariate statistics, identify biomarkers which distinguish case from control groups. There are a wide range of examples where non-targeted metabolomics has been employed in both the natural and life sciences, including assessment of complex interactions between diet and health[11,12], fingerprinting sub-lethal responses to environmental contaminants[13,14] and novel biomarker discovery associated with disease or xenobiotic exposure[15,16].

Among targeted approaches, native and isotopically-labelled standards facilitate metabolite identification and quantification, thereby reducing false positives which may lead to misinterpretation of an affected biological pathway. Quantitative metabolomics may be used to establish baseline metabolite levels in a tissue or organism for inter-laboratory comparison or for defining healthy versus ‘perturbed’ states. The use of isotopically labelled internal standards (IS) can also help to account for matrix-induced ionization effects which affect analytical precision, thereby enhancing sensitivity of the assay for detecting a biological response[17]. The major disadvantage of targeted approaches is limited coverage of the metabolome which increases the risk of overlooking the metabolomic response of interest.

In contrast to targeted metabolomics, non-targeted approaches offer the potential to determine novel biomarkers. However, this approach is never truly unbiased, since researchers must select a combination of stationary phase and ionization mode which may improve detection of some substances and reduce detection of others. The effect of these instrumental parameters on analytical coverage are poorly reported in the literature. False identification of metabolites or bias/signal drift introduced from matrix effects may also occur in non-targeted metabolomics due to a lack of standards. The latter may be corrected using any number of different signal-drift correction algorithms, but the relative performance of these approaches remains unclear. Finally, the lack of absolute quantification in non-targeted metabolomics hampers benchmarking of ‘normal’ metabolite levels and ultimately interlaboratory comparison of results.

In theory, the weaknesses encountered in one metabolomics approach are the respective strengths of the other. Nevertheless, there are few studies which have systematically compared the performance of targeted and non-targeted metabolomics[18]. The present work aimed to address this knowledge gap. Firstly, we developed independent targeted and non-targeted metabolomics platforms: The targeted approach involved (i) ultra-high performance liquid chromatography tandem mass spectrometry (UHPLC-MS/MS) for quantitative determination of 39 metabolites; and (ii) flow injection-tandem mass spectrometry (FI-MS/MS) for semi-quantitative analysis of 142 lipids. The non-targeted approaches were based on UHPLC-Orbitrap-mass spectrometry. Secondly, within the non-targeted approach, we assessed which combination of ionization mode (positive or negative) and stationary phase (C18 versus HILIC) produced the greatest analytical coverage (i.e. number of features). We then investigated the effect of a wide range of signal drift correction algorithms on the precision of non-targeted measurements as well as how well MS^2^ mass-library mzCloud performed in identifying compounds in the different sample matrices. Finally, we compared the precision of the optimized targeted and non-targeted methods for a limited number of substances detected by both approaches through analysis of replicate biological samples (including reference materials). This is among the few studies that have systematically evaluated the performance of targeted and non-targeted metabolomics and provides insight into the advantages and disadvantages of each approach.

## Materials and methods

### Metabolite nomenclature

A full list of targets and abbreviations, including Human Metabolome Database (HMDB) numbers, are provided in S1 Table. Briefly, for amino acids, neurotransmitters, and nucleobases, we adopted a standard 3-letter notation (e.g. alanine = ala, leucine = leu, etc). Glycerophospholipids were defined based on the presence of ester and/or ether bonds (represented by an ‘a’ or ‘e’, respectively), the length of fatty acid chains, and the number of double bonds. For example, lysoPCaC20:2 represents a phosphatidylcholine containing 1 fatty acid of 20 carbons in length, with 2 double bonds. Two letters (ae = acyl-alkyl, aa = diacyl) indicate fatty acids bound to two glycerol positions (e.g. PCaaC20:2). Sphingomyelin (SM) nomenclature includes a ‘d’ denoting the backbone sphingosine, with chain length and the number of double bonds separated by a colon, and a ‘C’ denoting the fatty acid, again with chain length and the number of double bonds separated by a colon (e.g. SM (d18:0/C18:1). Carnitines are denoted by a ‘C’ followed by the corresponding number of carbons and double bonds separated by a colon (e.g. C2:0). Note that this naming system, and the associated FI-MS/MS methods used herein does not identify the exact position of double bonds, nor the number of carbon atoms on different fatty acid side chains.

### Standards and reagents

Acetonitrile (ACN; HPLC grade) and chloroform (CHCl_3_) were acquired from VWR Chemicals (Radnor, PA, USA). Methanol (MeOH; HPLC grade) was acquired from Merck (Darmstadt, Germany). Water was filtered in-house to a total organic content < 3 ppb using a Milli-Q Integral 3 and a Millipak express 40 (0.22μm) filter (Millipore, Merck, Darmstadt, Germany). Native standards for amino acids, biogenic amines, neurotransmitters, and TCA intermediates were all of analytical grade and purchased from Sigma-Aldrich (St. Louis, Missouri, United States). Native carnitine and phospholipid standards (C2:0, PCaaC34:0, PCaaC28:2, lysoPCaC10:0, lysoPCaC24:0) were purchased from Avanti Polar Lipids (Alabaster, United States). The IS SMC16:0, L-methionine-methyl (^13^C, D_3_) and C0 (trimethyl-D_9_) were all acquired from Sigma-Aldrich (St. Louis, Missouri, United States). The remaining lipid ISs (lysoPCaC13:0, lysoPCaC17:1, PCaaC25:0, PCaaC31:1, PCaaC43:6 and SMC12:0) were acquired from Avanti Polar Lipids (Alabaster, United States). ZrO (2.0 mm) and stainless steel beads (4.8 mm) were purchased from Next Advance (New York, United States).

Native amino acids, biogenic amines, nucleobases and neurotransmitters were prepared in a mixture of filtered Milli-Q water and MeOH (60:40) containing 1% (v/v) formic acid (FA), while carnitines and lipids were prepared in 100% MeOH. The IS used for amino acids, biogenic amines, nucleobases and neurotransmitters was solved in 20:80, MilliQ and MeOH with 1% (v/v) FA addition. The ISs for carnitines and lipids were solved in pure MeOH.

A stock solution of native amino acids, biogenic amines, nucleobases and neurotransmitters was diluted into 11 calibration standards ranging from 346.3 pM to 161.2 μM of individual targets (S2 Table). Final IS concentration in samples and standards for the QqQ_HILIC_ analysis was 0.19 μM while the five standards used for QqQ_FI_ analysis ranged from 0.11 to 0.57 μM (S2 Table).

### Plasma and tissue samples

The fish processing was carried out in careful accordance to the rules of the Local Authority and evaluated and approved by the Bioethics Committee of the University of the Basque Country (UPV/EHU) (CEEA/380/2014/ETXEBARRIA LOIZATE). Post termination and dissection, the brain and liver samples were stored at -80°C, shipped on dry ice to Stockholm University where they were once again stored at -80°C prior to analysis. Standard Reference Material (SRM) 1950, *Metabolites in Human Plasma*, was obtained from the National Institute of Standards and Technology (NIST). Upon receipt, the material was aliquoted into 12 vials (100 μL each), and then stored at -80°C prior to use. The NIST material was extracted in two batches together with gilthead bream brain or liver tissue, at two different time points: the first and second batch containing four and eight replicates, respectively. Detailed sample information is available in S3 Table.

### Workflow

**Fig 1:**
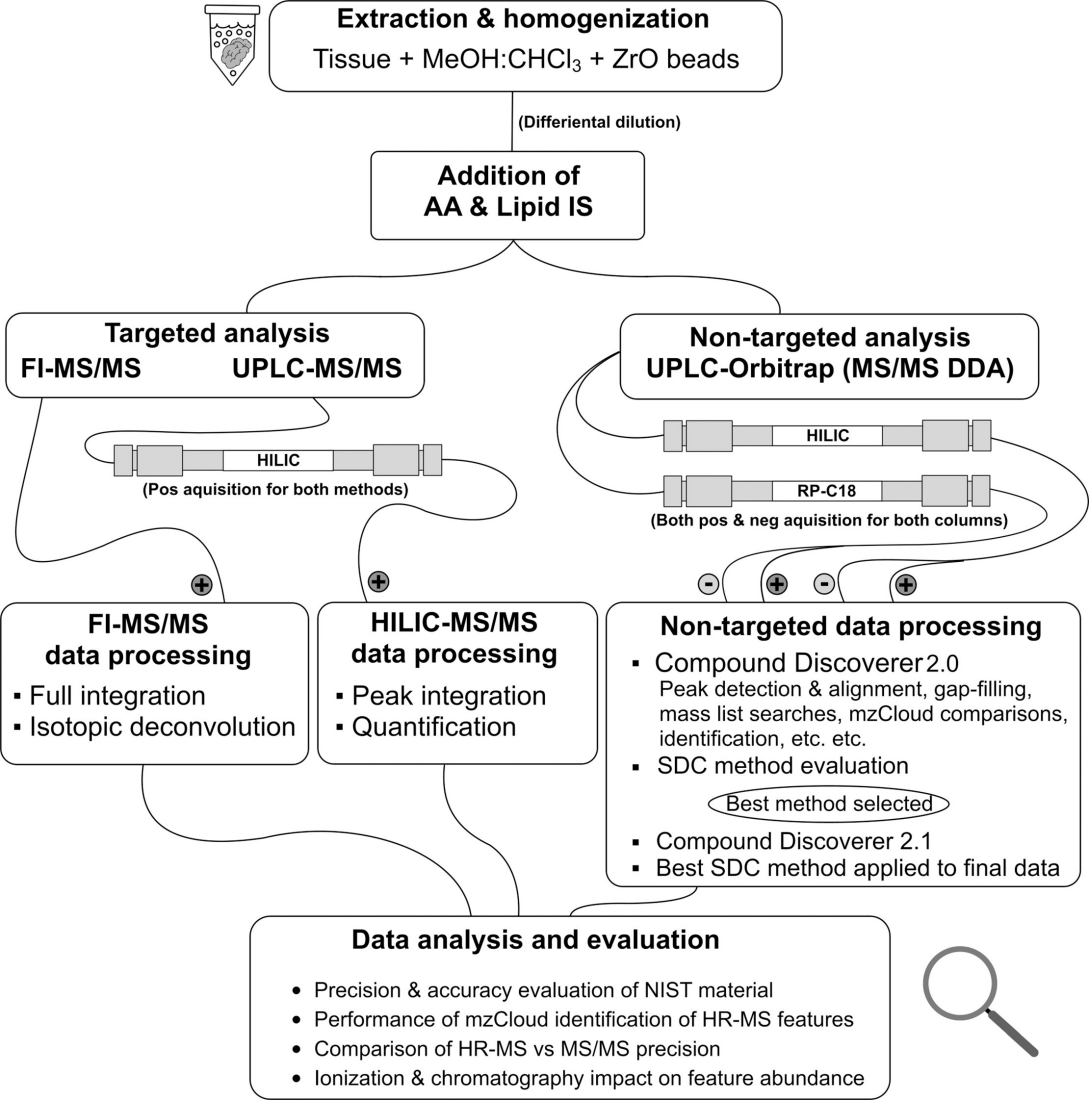
Study workflow. An overview of the workflow adapted for the analytical method, the processing required post acquisition and the analysis and evaluation of the collected data. Hydrophilic interaction chromatography (HILIC), Flow-Injection (FI), Signal Drift Correction (SDC).

The workflow of this study is outlined in Fig 1. The first step involved analysis of three sample types (NIST human plasma, fish liver and fish brain) using 3 different analytical approaches; UPLC-HILIC-MS/MS (QqQ_HILIC_), FI-MS/MS (QqQ_FI_) and UPLC-Orbitrap MS (Orbi_HILIC/C18+/-_). The QqQ_HILIC_ and QqQ_FI_ analyses (both targeted) consisted of acquisition followed by quantitation or semi-quantitation with isotopic deconvolution, respectively. Accuracy was assessed by comparing measured concentrations in NIST human plasma to reference concentrations while precision was assessed as the relative standard deviation (RSD) of replicate plasma and tissue samples. Non-targeted analysis involved optimization of peak-picking settings, after which 7 different signal drift correction methods were evaluated. The signal drift correction method yielding the lowest RSD was used for comparing the precision of targets in the non-targeted dataset to those detected by the targeted methods. Finally, the accuracy of the online MS2-database “mzCloud” was evaluated using standards of 38 different metabolites. All scripts utilized in data processing were programmed in the statistical software R[19] and compiled into a package called ‘metLab’ which is available at github[20].

### Sample extraction

Extraction efficiency experiments revealed that CHCl_3_:MeOH (20:80) was the most effective solvent for extraction of metabolites targeted in the present work. Brain sample extraction (~100 mg) was initiated by adding tissue, 5 μL CHCl_3_:MeOH (20:80) per mg tissue and ZrO beads (2.0 mm), into 1.5 ml polypropylene (PP) tubes. For the liver samples (~0.5-1g), 13 ml PP tubes and 4.8 mm stainless steel beads were employed, using the same solvent to tissue ratio as for brain samples. All samples were homogenized for 4 min at 1500 rpm, using a 1600 MiniG homogenizer (Spex Sample Prep, New Jersey, United States). Brain samples were centrifuged using a Galaxy 14D microcentrifuge (VWR, Radnor, PA, USA) for 10 minutes at 8100 g while liver samples were centrifuged in a Eppendorf 5804 R (VWR) equipped with a F-34-6-38 fixed-angle rotor (Eppendorf, VWR) for 10 minutes at 12900 g. Two dilutions per tissue, 1:5 and 1:100 for brain and 1:15 and 1:300 for liver, were carried out with pure MeOH before addition of 120 μL of IS solution (>99% MeOH). Extraction of human plasma was carried out by adding 500 μL of CHCl_3_:MeOH (20:80) and ZrO beads (2.0 mm) to PP-tubes containing the 100 μL aliquots of sample. The samples were homogenized for 4 min (1500 rpm) and then centrifuged (8100 g) for 10 minutes using a Galaxy 14D microcentrifuge (VWR, PA, USA). Prior to analysis the samples were diluted 1:5 through addition of 120 μL of IS solution (>99% MeOH) to 30 μL of NIST extract.

### QqQ_HILIC_-analysis

Targeted analysis of amino acids, biogenic amines, nucleobases and neurotransmitters was carried out using a Dionex UltiMate 3000+ coupled to a TSQ-Quantiva (Thermo Scientific, Waltham, Massachusetts, United States) via a heated electrospray ionization (HESI) source. Targets were chromatographed on an Acquity BEH-Amide hydrophilic interaction liquid chromatography (HILIC) column (2.1 × 100 mm, 1.7 μm) equipped with a Vanguard pre-column (2.1 × 5 mm, 1.7 μm) from Waters (Milford, Massachusetts, United States). The mobile phases consisted of (A) Milli-Q water with 0.1% FA and (B) ACN with 0.1% FA. The eluent flow rate was maintained at 0.2 ml/min from 0–2.5 min, then increased to 0.5 ml/min from 2.5–2.8 min, at which point it was held constant until 9.5 min, then increased again to 0.8 ml/min from 9.5–9.8 min where it was maintained until 10.8 min. The flow rate was then reduced from 0.8–0.2 ml/min from 10.8–11.2 min where it was maintained until the end of the run (11.3 min). During this time, the gradient of the mobile phase was ramped as follows: 0–2.8 min, 3% A; 2.8–6 min, 30% A; 6–8.5 min, 60% A; 8.5–9.5 min, 60% A; 9.5–11.3 min, 3% A. The column was kept at 30°C throughout the analysis while samples in the auto-sampler were kept at 5°C. Injection volume was 2 μL of extract and the washing program consisted of flushing the needle with 50 μL of 90:10 (H_2_O:MeOH) before and after every injection. Source voltage was 5000V in positive ion mode and 4500V in negative ionization mode, sheath gas was 45 (arbitrary units), auxiliary gas was 20 (arbitrary units), sweep gas was 0.5 (arbitrary units), source fragmentation was 20 V, CID gas pressure was 1.5 mTorr, while ion transfer tube and vaporizer temperature both were set to 350°C. The instrument was set to multiple reaction monitoring (MRM) using the transitions and radio frequencies (RFs) listed in S1 Table and the instrument parameters listed in S4 Table. Acquisition was carried out using XCalibur 3.1 (Thermo).

UPLC-MS/MS data were processed using XCalibur 3.0.63 (Thermo). For each target, a calibration curve with at least 5 points was generated with concentrations ranging from 3.5 nM– 151.5 μM. Most metabolites were fit to linear calibration curves with 1/x^2^ weighting. The exceptions were creatinine, malic acid and taurine, which were fitted to quadratic curves with log-log weighting. The correlation coefficients for the calibration curves ranged between 0.946 and 0.999 (S2 Table). Detection limits were defined as the signal-to-noise ratio calculated by XCalibur for the lowest calibration standards in which a peak was still measurable, divided by itself and multiplied by three (S2 Table).

### QqQ_FI_ analysis

Analysis of carnitines and lipids was carried out using a method adapted from Liebisch et al. 2005[21,22]. PEEK tubing (120 cm, 0.005” ID, 1/16” OD) was connected directly between the UHPLC and the MS (i.e. a chromatography column was not used). The mobile phase consisted of MeOH:CHCl_3_ (3:1) containing 10 mM ammonium acetate[21] which was maintained at isocratic conditions throughout the run. The flow rate was ramped as follows: 0.0–0.4 min, 0.065 ml/min; 0.4–0.5 min, 0.065–0.030 ml/min; 0.5–1.5 min, 0.030 ml/min; 1.5–1.6 min, 0.030–0.090 ml/min; 1.6–5.0 min, 0.090 ml/min; 5.0–5.1 min, 0.090–0.50 ml/min; 5.1–5.7 min, 0.50 ml/min; 5.7–6.0 min, 0.50–0.065 ml/min. An exhaustive needle cleaning method was required to prevent carry-over using this method. The procedure involved a 20 μL draw and discharge of isopropanol, CHCl_3_ and MeOH (in that order) before sample injection. Samples were maintained at 5°C in the auto-sampler and the injection volume was 10 μL. Optimization of the HESI-probe resulted in settings of 3600 V for source voltage (+HESI), 15 for sheath gas (arbitrary units), 7 for auxiliary gas (arb), sweep gas 0 (arbitrary units), 25 V for source fragmentation, and 1.5 mTorr for CID gas. Ion transfer tube and vaporizer temperature were set to 325°C and 110°C respectively. As with the QqQ_HILIC_ method, acquisition was carried out in MRM-mode, using the transitions and RFs stated in the supplementary information (S1 Table) with XCalibur 3.1 (Thermo) and the instrument parameters listed in S4 Table.

Peak integration was carried out using XCalibur 3.0.63. Automated peak integration using XCalibur lead to a large proportion of lipids with either partially, or non-integrated peaks. Consequently, the mouse macro-script software ‘Macro recorder’ (Jitbit LP, Edinburgh, United Kingdom) was employed for semi-automated full chromatogram integration (Text A in S1 Appendix). Peak areas were then subjected to isotopical deconvolution using the “fullDecon” function in our R-package, which is a version of the Liebisch et al. deconvolution method [21]. The deconvoluted data was then exported to Excel (Microsoft, Redmond, Washington, USA) for quantification, carried out according to Eq 1. ISs for individual lipids are stated in S2 Table. Background levels were negligible relative to the signal produced by the IS. Limit of detection and quantification were determined as 10 times the concentration in the blanks run with liver samples (S2 Table).

$$\frac{{Area}_{analyte}}{{Area}_{IS}}*{Conc}_{IS}*Dilution\;factor={Conc}_{analyte}$$ (1)

### Orbi_HILIC/C18+/-_ analysis

Non-targeted analysis was carried out using a Dionex Ultimate 3000+ coupled to a Q Exactive HF Hybrid Quadropole-Orbitrap MS (Thermo Scientific, Waltham, Massachusetts, United States). Two separate chromatographic methods were employed, in both positive and negative mode, for a total of four analytical methods. The first method involved the same HILIC column and mobile phase as was used for the QqQ_HILIC_ analysis, with a slightly modified gradient. Initial conditions were 3% A / 97% B, which were maintained from 0–1.5 min, then ramped to 25% A by 8 min, 40% A by 9.5 min, and 60% A by 11 min. By 13 min, the gradient was returned to initial conditions (3% A), then equilibrated for a further 0.9 min prior to the next injection. The flow was changed throughout the analysis as follows: 0–13 min, 0.3 ml/min; 13–13.2 min, 0.3–0.6 ml/min; 13.2–13.7 min, 0.6 ml/min; 13.7–13.9 min, 0.6–0.3 ml/min. The second method employed an Acquity UPLC HSS T3, C18 column (2.1 × 100mm, 1.8 μm) equipped with a Vanguard pre-column (2.1 × 5 mm, 1.8 μm) from Waters (Milford, Massachusetts, United States). The mobile phases were a modified version of the setup used by Hu et al.[23]; A: Milli-Q + 10 mM ammonium formate (AF) and B: isopropanol:ACN (90:10) + 10 mM AF. The same mobile phases were used for both positive and negative analysis since weak acids have been shown to be favorable for both types of ionization[24]. For both ionization modes the following gradient was used: 0–1 min, A(98%), B(2%); 1–7 min, A(70%), B(30%); 7–15 min, A(2%), B(98%); 15–17 min, A(98%), B(2%); 17–19 min, A(98%), B(2%); 19–20 min, A(98%), B(2%). The flow gradient was altered during analysis as follows: 0–16 min, 0.25 ml/min; 16–16.1 min, 0.25–0.4 ml/min; 16.1–17.1 min, 0.4 ml/min; 17.1–17.2 min, 0.4–0.25 ml/min; 17.2–19.0 min, 0.25 ml/min.

All samples were analyzed using both Orbi_HILIC_ and Orbi_C18_ methods, in both positive and negative modes, resulting in 4 analyses per sample. For each analysis, the Orbitrap was set to full scan with data-dependent MS^2^ (Full MS→ddMS^2^), where the full scan (*m/z* 70–1000) had a resolution of 120,000 and a maximum injection time (IT) of 200 ms. For every full scan, three ddMS^2^-scans were carried out with a resolution of 15,000, a minimum AGC (automatic gain control) target of 3.00×10^3^ or 8.00×10^3^ for Orbi_HILIC_ or Orbi_C18_ analysis respectively, a maximum IT of 30 ms and a stepped normalized collision energy (NCE) of 10, 20 and 35 (eV). All data were acquired in profile mode using XCalibur 3.1.66.10. The ESI tune-file for positive mode had the following settings: Source voltage 3700V, sheath gas 28 (arb), aux gas flow rate 7 (arb), sweep gas flow rate 1 (arb), S-lens RF level 55.0 and both the capillary and aux gas heater temperatures set to 350°C. The ESI tune-file for negative mode was the same as for positive with the exception of sheath gas flow rate which was set to 25 (arb). The mass-calibration solutions used for positive and negative mode were the Pierce LTQ ESI Positive Calibration Solution and Pierce LTQ ESI Negative Calibration Solution from Thermo (Waltham, Massachusetts, United States). For mass-calibration of the instrument, we used a custom list which included lower masses than the default calibration provided with the instrument (see S4 Table for all information regarding instrumental settings of Orbitrap analysis). After positive and negative calibration, the root mean squared mass accuracy was 0.15/0.21 ppm and 0.24/0.33 respectively.

Orbitrap data were processed using CD 2.0 (sequence correction) or 2.1 (all other data processing) (Thermo-Fisher Scientific, USA). The full CD-workflow and settings are found in S1 Description. Criteria for putative identification were chosen as a combination of two different properties; an mzCloud match score higher than 90% and the same identification being suggested by at least one of either BioCyc, HMDB, Kyoto Encyclopedia of Genes and Genomes (KEGG), LipidMaps, Pubchem or Small Molecule Pathway Database (SMPDB).

### Signal drift correction

A variety of quality control (QC) samples were included to carry out and evaluate sequence and inter-batch signal drift corrections. These included, a) technical replicate sequence QC injections used for training the drift models (QC_train_) and b) extraction batch QCs used to test the performance of the correction (QC_test_). We tested four different methods to counteract signal drift and batch effects during non-targeted analysis. Three approaches used sequence and/or extraction batch QCs: linear sequence correction[25], the batch MetaboAnalyst 3.0 correction feature[26] and batchCorr[27]. The fourth approach involved simply dividing metabolite peak areas by the peak area of the IS (i.e. IS correction). Together, these four methods and three combinations thereof (i.e. a total of seven different approaches) were evaluated for their potential to reduce RSDs of selected metabolites in the NIST reference material. We also evaluated the potential of each method to increase the number of features displaying RSDs of less than 15 or 30% in QC_train_ and QC_test_.

### Evaluation of method performance

Accuracy of the QqQ_HILIC_ method was assessed by comparing measured concentrations of 12 metabolites in NIST serum to certified concentrations and literature. For the QqQ_FI_ method the accuracy was evaluated through comparisons with literature. Method within- and between-batch precision as well as technical replicate precision were assessed using replicates of serum and tissue samples analyzed within and between batches.

For the non-targeted workflow, three performance metrics were evaluated: 1) the potential of the method to correctly identify metabolites, evaluated by comparison to substances measured in the targeted workflow; 2) precision of the non-targeted approach, as compared to targeted analysis; and finally, 3) an evaluation of the number of features obtained by Orbi_HILIC_ versus Orbi_C18_ in both positive and negative modes. The first metric was assessed by counting the number of compounds passing our putative identification filter (i.e mzCloud score >90% + exact mass library match). We also assessed the factors controlling the success of mass spectral library matching using mzCloud by processing non-target data both with and without inclusion of reference standards (i.e. the same standards used for QqQ_HILIC_). The second metric was evaluated by comparing RSDs for a subset of metabolites detectable by both the targeted and non-targeted methods. In order to compare the precision of lipids measured via QqQ_FI_ with those measured by Orbi_C18+_, a lipid search script was designed (part of our R-package, see Text B in S1 Appendix for details) which flags lipids from the non-target dataset. This script is necessary because the current version of CD does not offer a high throughput option for linking many MS^2^ fragments to their parent ions. Our newly developed script obtains the retention time for a given lipid fragment (e.g. *m/z* 184 for PCs) and then matches this to the parent ion at the same retention time. Finally, the third metric was evaluated through comparison of the total number of features acquired from CD 2.1 processing of all non-target data.

## Results and discussion

### Performance of targeted approach

**Fig 2:**
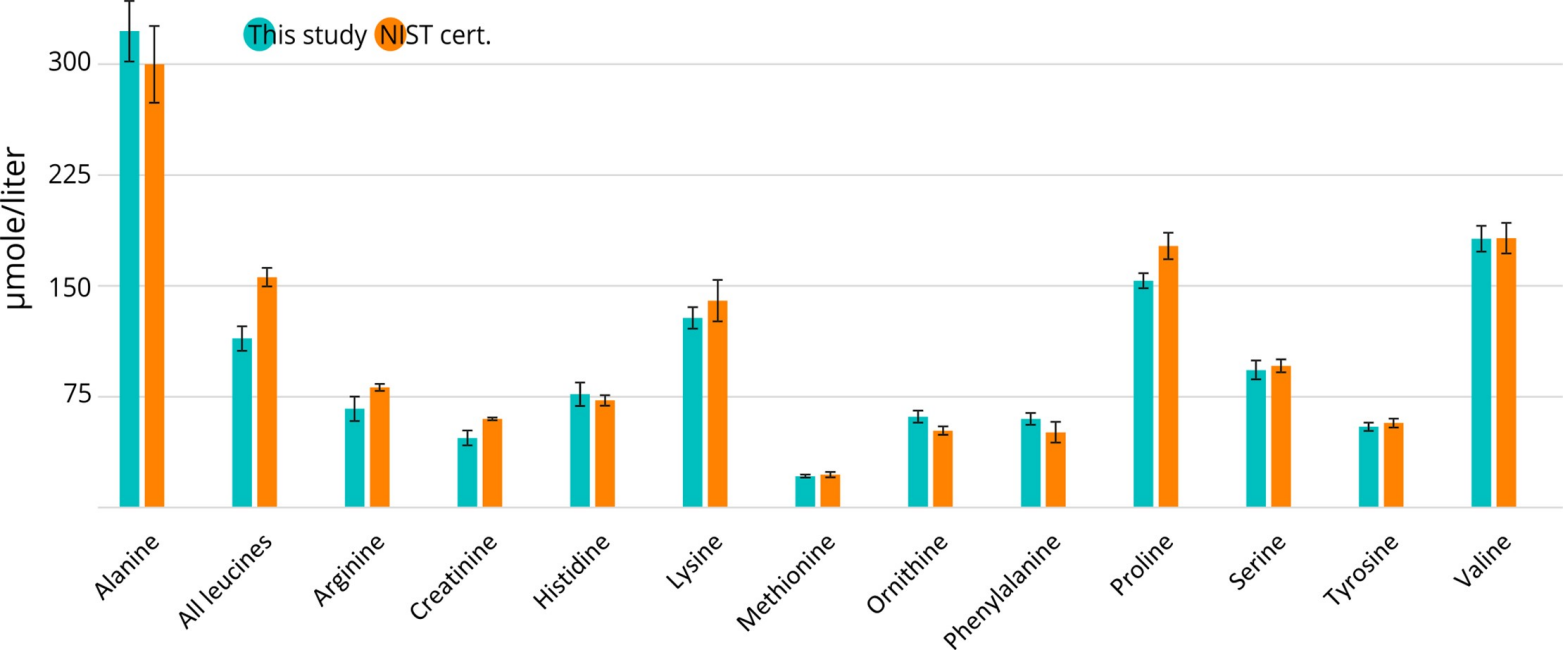
Accuracy of the QqQ_HILIC_ method evaluated through comparison with the NIST SRM 1950 certificate[28]. Error bars represent standard deviation for blue bars but ‘expanded uncertainty’, as described in the NIST certificate for SRM 1950 for orange bars [28].

A total of 90 of the 181 targeted metabolites (67 lipids / 23 non-lipids) were detectable in NIST plasma. For targets measured by QqQ_HILIC,_ method accuracy ranged from 73–119% (n = 12 metabolites) when compared to reference values for NIST plasma[28] (Fig 2) while RSDs ranged from 3.3 and 18.7%, (average 7.8%). This is particularly impressive considering the few internal standards used in the method; albeit inclusion of additional internal standards may improve accuracy and precision beyond that reported here. For lipids measured by QqQ_FI_, NIST reference values were unavailable. A comparison of lipid data for SRM 1950 revealed that concentrations for up to 33 lipids measured in the present work were within 50–150% of those reported by either Quehenberger et al. or Bowden et al. [29,30]. For the remaining lipids measured in our study, concentrations were either outside this range or were not reported. Thus lipid data reported here should be considered semi-quantitative. Notably, all lipids displayed excellent precision, ranging from 3.7–28.2% (mean 10.9%), which is comparable to the precision of non-lipidic metabolites measured by HILIC. Considering the lack of chromatography and few standards used in the QqQ_FI_ approach, the performance of the method is reasonable. It is germane to note that some inconsistencies exist between previously published data for lipid concentrations in SRM 1950. For example, Quehenberger et al. reported 16.2 μM of SM(d18:0/C18:0) while Bowden et al. reported 2.0 μM [29,30]. Clearly, further work is necessary to develop reliable reference concentrations for lipids in SRM 1950.

In fish tissues, 122 of 181 targets were detectable in liver (92 lipids and 30 non-lipids) and 130 of 181 targets (101 lipids and 29 non-lipids and) were detected in brain. Accuracy could not be assessed in these tissues due to a lack of a certified reference material or ‘metabolite-free’ tissue necessary for performing realistic spike/recovery experiments. Nevertheless, precision in both tissues (regardless of metabolite) was excellent, with average RSDs of 6.1 and 8.0% for lipids and non-lipids in liver (n = 15), respectively, and 6.4 and 8.4% for lipids and non-lipids in brain (n = 11) (S5 Table).

### Performance of non-targeted approach

**Fig 3:**
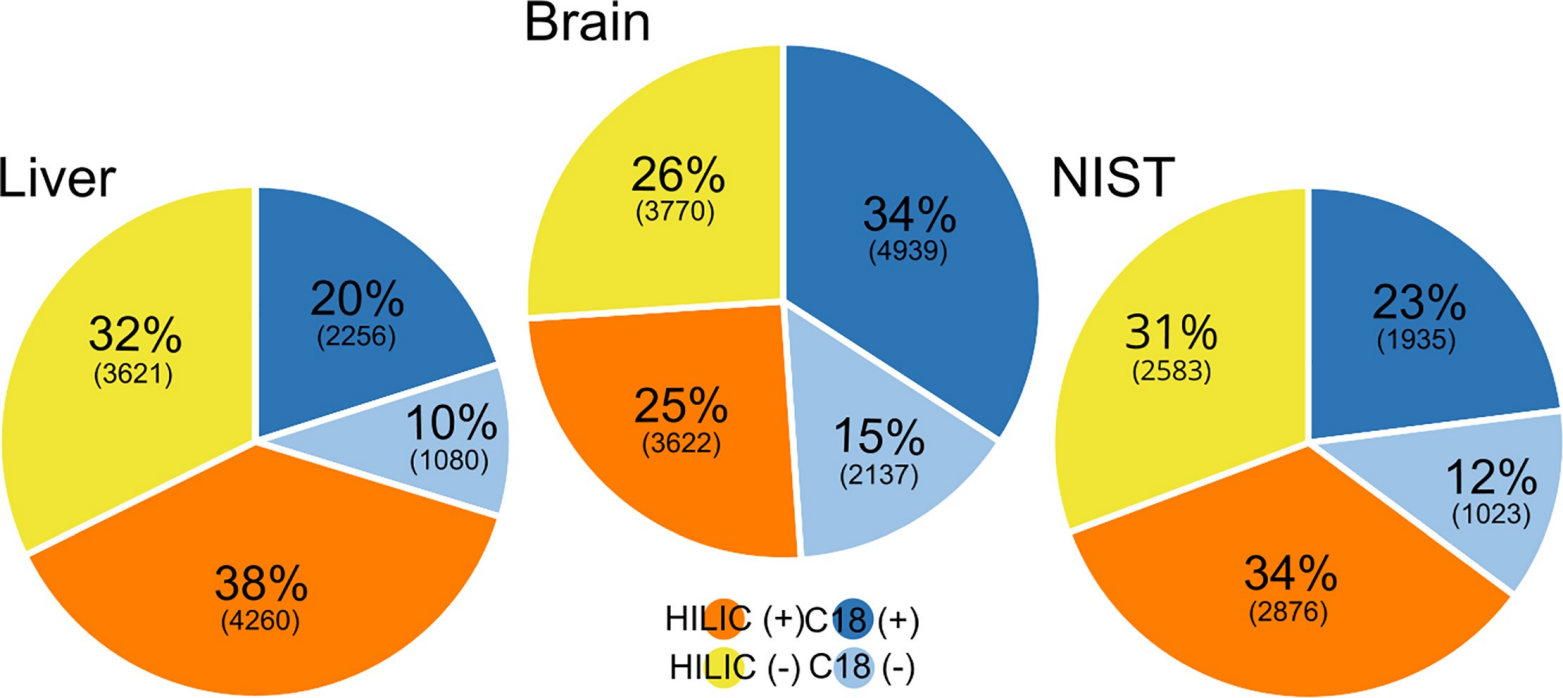
The impact of chromatography and ionization on the number of features obtained from the same samples run through four different combinations of chromatography and ionization (C18(+), C18(-), HILIC(+) and HILIC(-)). Portrayed here as the percentage of each different combination of the total summed amount of features detected in the same sample type and the number of features within parentheses (S6 Table).

Peak-picking during non-target analysis required manual optimization and evaluation before it was applied to the non-target dataset. Optimized parameters included dataset composition (i.e. type of samples included in a batch processed by CD), signal intensity thresholds, retention time and mass windows. The optimized non-target workflows were then applied to each dataset produced from a given tissue (i.e. analysis by C18 and HILIC columns in both ionization modes) to investigate which chromatography/ionization combination detected the most features. The results for each tissue are summarized in Fig 3 (Detailed breakdown in S6 Table). Overall, analysis using +HESI resulted in 133–145% more features compared to–HESI (sum of all features in both C18 and HILIC), highlighting the effectiveness of this ionization mode for maximizing analyte coverage. HILIC produced 104–236% more features than C18 based on the sum of all features in positive and negative mode in the same tissue. Therefore, in the absence of *a priori* hypotheses necessitating a particular ionization approach/chromatographic method (e.g. interest in fatty acids could prompt the use of C18 in negative mode), a +HESI/HILIC analysis is expected to produce the largest number of chromatographic features.

**Fig 4:**
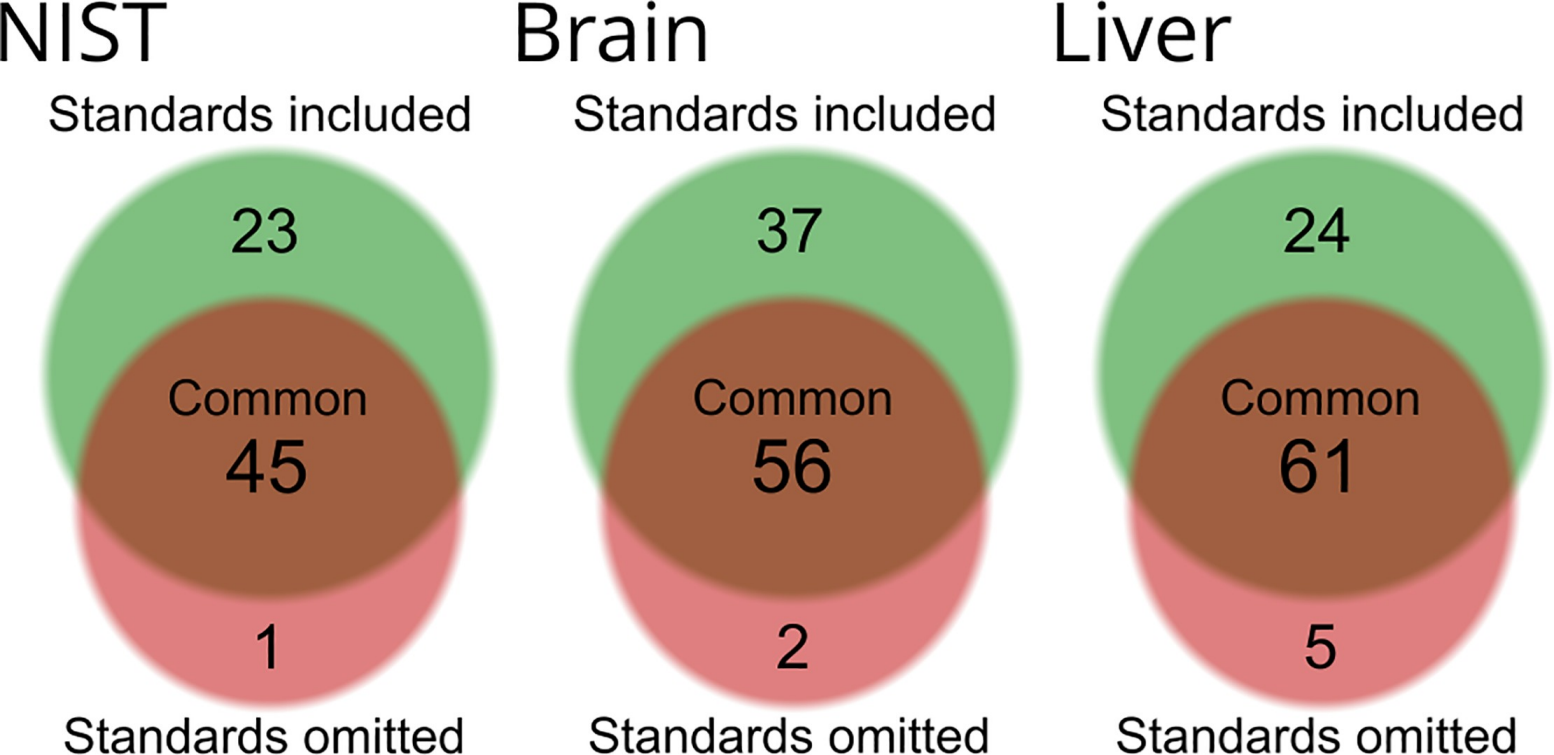
**Number of compounds living up to our mzCloud filter-criteria in NIST (left), brain (center) and liver (right).** Compounds are grouped based on how many compounds were detected through CD analysis with (green) or without reference standards (red). Any compounds which were only detected in the reference standards were removed from these graphs.

The results from the mzCloud identification performance tests are provided in Fig 4 (See S1 Dataset for details). For all tissues, the datasets processed together with reference standards had a higher number of features living up to our mzCloud matching criteria. In brain, 85 features were identified when reference standards were included in data processing, while only 58 were identified when standards were not included. A similar finding was made in liver (i.e. 83 identified when processed with standards versus 66 without) as well as in the NIST material (68 identified with standards versus 46 without).

In mzCloud, the similarity between a substance’s library MS^2^-spectrum and the single best-quality MS^2^-spectrum in the experimental dataset determines its mzCloud score. In assigning this score, mzCloud assumes that the best-quality MS^2^-spectrum acquired from one sample is representative of the MS^2^-spectra for all other samples, thereby relying on retention time and exact mass of the parent ion to ensure the substance is the same across all samples. This can be problematic if the substance producing the best-quality MS^2^-spectrum is not the same as in the other samples (e.g. in the case of isomers). The problem may be exacerbated when including standards in the data processing workflow, since the MS^2^-spectrum of the standard will undoubtedly be selected over that of samples. In the present work, the number of features passing our putative identification filter increased by an average of 40% in all three tissues when reference standards were included and we confirmed that there were no instances of misidentification. Nevertheless, caution is warranted when interpreting mzCloud scores in suspect-confirmation analysis if reference standards and samples are processed in the same CD analysis. Judging by these results, a compound living up to the criteria of our filter in a dataset comprised solely of samples is most likely to be assigned level 2 putative identification[31]. This means that when faced with the decision whether to invest resources into confirming the identity of a compound of interest, passing the filter is a strong indicator of that this is plausibly worthwhile.

### Signal drift modelling and correction of non-target workflow

**Fig 5:**
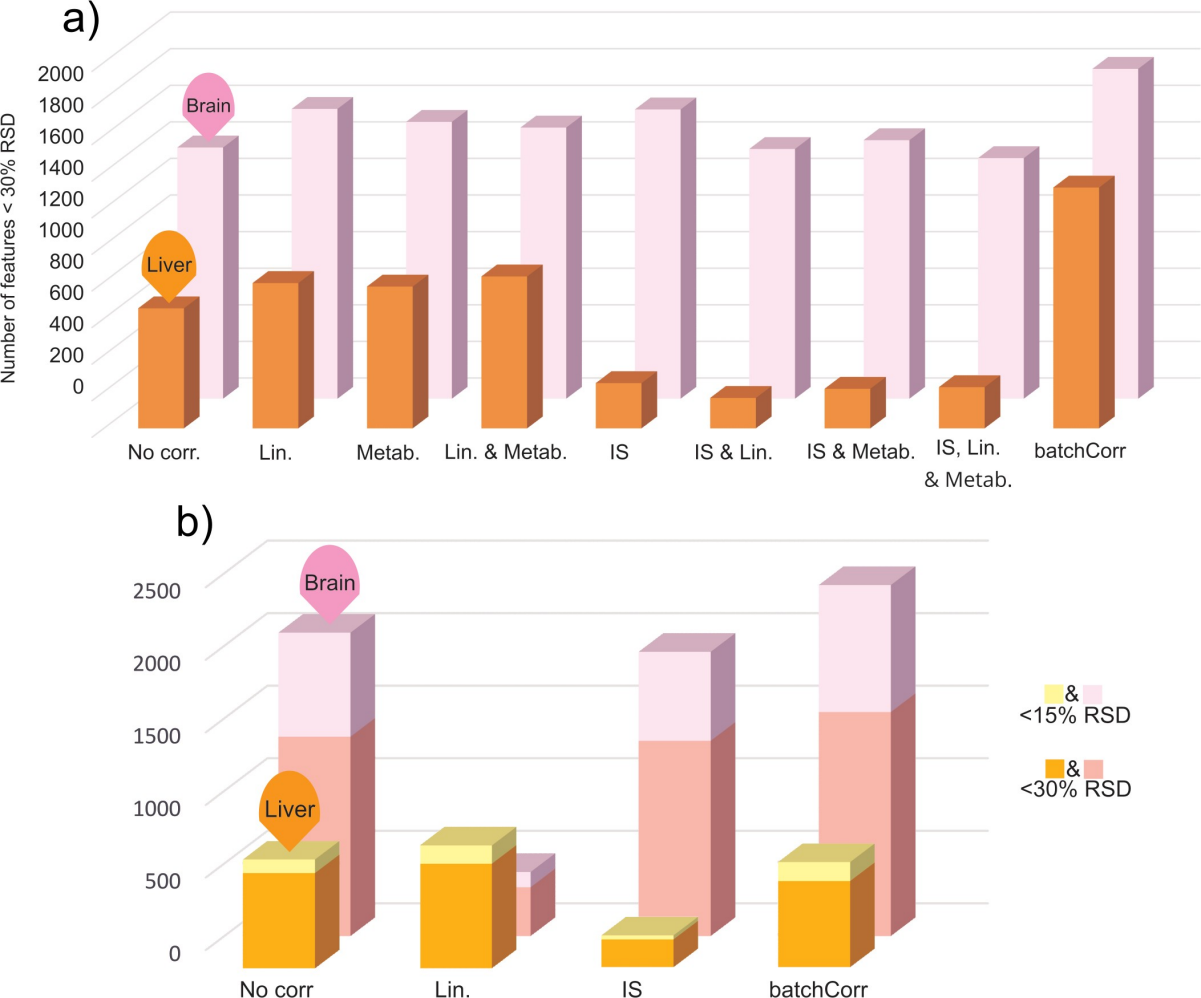
Comparison of different signal drift correction techniques in QC_test_ & QC_train_. a) Comparison in terms of how many features that were below 30% RSD in QC_train_. The acronyms used for the different signal drift methods are as follows: “No corr.” = No correction; “Lin.” = Linear sequence correction; “Metab.” = Metaboanalyst batch correction; “IS” = Internal standard correction; “batchCorr” = batchCorr. b) Number of compounds in brain and liver QC_test_, below 30% and 15% RSD post correction.

The performance of each of the 4 sequence correction methods, and combinations thereof, is summarized in Fig 5 (S2 Dataset for details). As shown in Fig 5, batchCorr outperformed the other signal drift correction methods in all comparisons except for in QC_test_ where linear sequence correction outperformed it in liver tissue. This is impressive considering that no adjustment to the run-order or QC-frequency requirements were made prior to analysis (i.e. batchCorr specifies injection of QC_train_ every 5th injection[29], while the present work used every 10th injection). Thus, batchCorr likely underperformed in this particular analysis despite having outperformed the other sequence correction cases in almost all cases. Therefore, batchCorr was applied to all non-targeted data as part of the data processing prior to comparison to the targeted data.

### Comparison of targeted versus non-targeted approaches

While triple-quadrupole instruments are often perceived as the gold standard for targeted metabolomics[3,6,17], this platform is not infallible and requires close scrutiny. In the present work, the application of Orbi_HILIC_ was used to identify two instances of false positives (serotonin and DOPA) in data generated through QqQ_HILIC_. In both cases, a closer inspection of HRMS data revealed features within ±0.5 Da of DOPA and serotonin, and with MS^2^ fragments consistent to those used for targeted analysis. However, when a narrower mass range was applied, these substances were not observable in samples. For the remaining targets, peaks observed in the QqQ_HILIC_ analysis were consistent with the Orbi_HILIC_ dataset.

**Fig 6:**
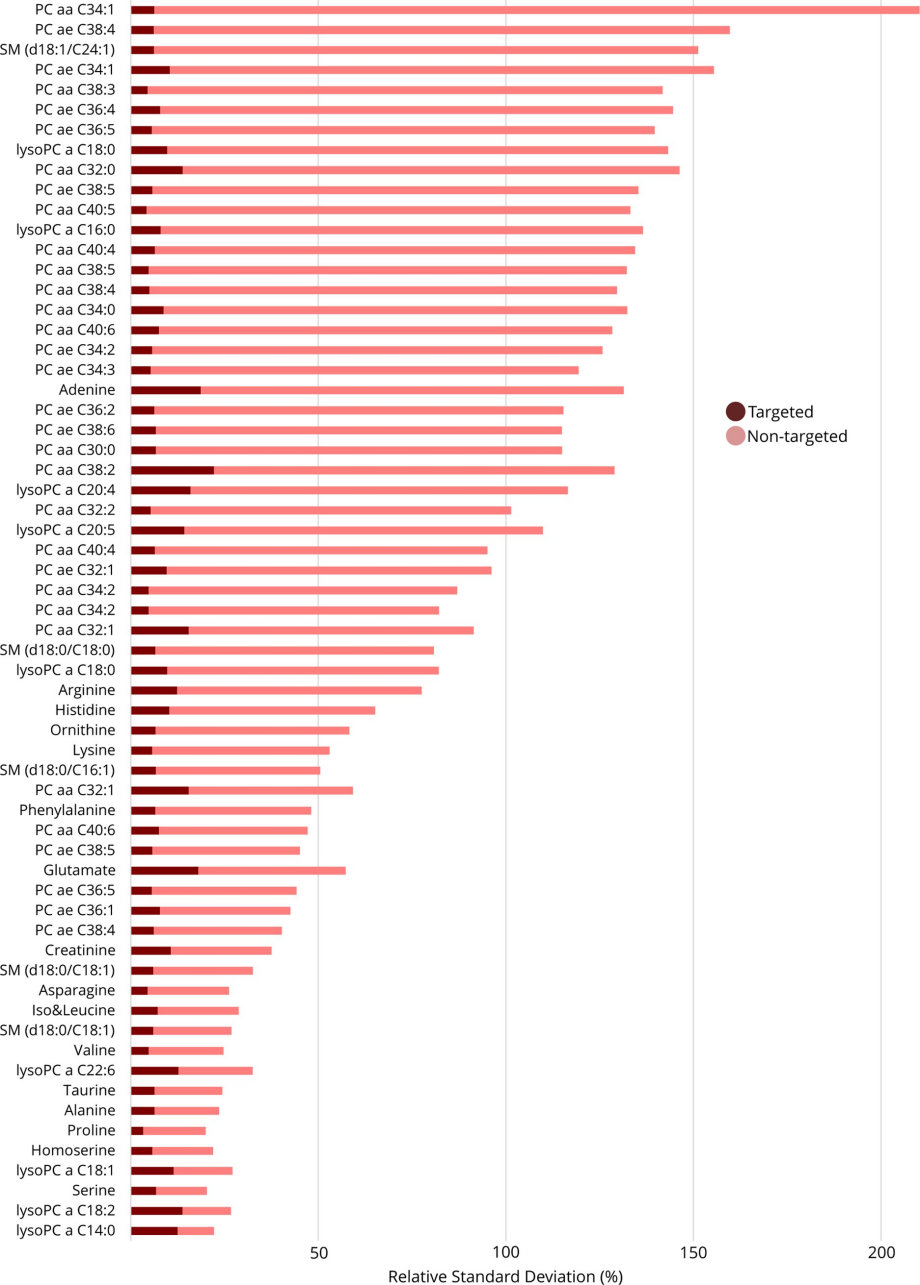
Targeted vs non-targeted precision in NIST SRM 1950. Features from Orbi_HILIC_ and Orbi_C18_, selected manually or using the ‘lipidFrag’ function in our R-package, compared to features measured with QqQ_HILIC_ and QqQ_FI_. Isomers or lipids from the low resolution QqQFI-method show up as duplicates in the list of compounds in the figure.

Analysis of the NIST material revealed 16 compounds comparable between QqQ_HILIC_ and Orbi_HILIC_ while the R-script filtered out 45 lipids from the Orbi_C18+_ method which were comparable to the QqQ_FI_ method. All of the non-lipid analytes had better RSDs in the targeted dataset versus the non-targeted (signal drift-corrected) dataset, as seen in Fig 6 (S3 Dataset). For the lipids, 44/45 of the analytes had better RSDs when analyzed using QqQ_FI_ (8.7% RSD) as compared to Orbi_C18+_ (92.1% RSD; S4 Dataset).

Analysis of liver revealed 21 targets measured by both QqQ_HILIC_ and Orbi_HILIC+_. For 19 of 21 substances, precision was higher when using QqQ_HILIC_ (mean 6.9% RSD) versus Orbi_HILIC+_ (mean 17.1% RSD) for technical replicates while for between-batch samples all 21 were superior in QqQ_HILIC_ (means RSDs of 4.7% and 70.8%, respectively; S3 Dataset). In total 36 lipids were comparable between QqQ_FI_ and Orbi_C18+_. Precision for these lipids was better for 34 of them in QqQ_FI_ versus Orbi_C18+_ considering technical replicates (mean 3.9% vs 30.2% RSD) and 35 when considering between-batch samples (mean 6.8% vs 33.3% RSD)(S4 Dataset).

Finally, analysis of brain tissue revealed 103 comparable substances (between QqQ and Orbitrap). Of the 22 non-lipids detectable by both platforms, 17 compounds had higher precision in QqQ_HILIC_ vs Orbi_HILIC+_ when considering technical replicates (mean 6.8% vs 19.9% RSD) while only 12 were better when considering between-batch samples (mean 11.9% vs 26.6% RSD; S3 Dataset). In technical replicates, 77 lipids had lower RSDs in QqQ_FI_ vs Orbi_C18+_ (mean RSDs of 7.0% vs 31.8%, respectively). For between-batch samples 77 lipids had higher precision in QqQ_FI_ vs Orbi_C18+_ which is also reflected in the lower average RSD of QqQ_FI_ compared to Orbi_C18+_ (mean 7.4% vs 36.4%, respectively) (S4 Dataset).

Overall, this comparison revealed that both targeted approaches (i.e. QqQ_HILIC_ and QqQ_FI_ analysis), have an overall higher precision than Orbitrap-analysis when configured as in this study. This difference is likely to stem from inherent challenges with data acquisition and processing of Orbitrap data, rather than technical superiority of QqQ methods. For example, the current version of CD (software used for processing Orbitrap data) does not include options to control or optimize peak integration, resulting in variable quality in peak integration. See Figure A and B in S1 Description. As Orbitrap data processing software improves, an improvement in the precision of non-target data can be expected. Nevertheless, at this point in time, our data indicate that a targeted methodology is more likely to detect a statistically significant perturbation due to substantially lower RSDs, provided the metabolites of interest are known.

## Conclusions

In this study we developed stand-alone targeted and non-targeted metabolomics approaches and then evaluated their respective performance through comparison to each other. Among the important findings of this study were that: (i) Targeted analysis has an overall better precision than non-targeted analysis (even after sequence signal drift correction). This is most probably due to non-optimal peak picking and integration in CD and will likely improve as the methodology behind processing Orbitrap data develops. (ii) Signal correction comparisons are non-trivial and in some cases yielded dichotomous results; nevertheless, for the purposes of retaining the most number of features, batchCorr outperforms other correction algorithms in most instances. (iii) Although mzCloud shows considerable promise as a tool for peak identification, it still has a long way to go before it will be able to positively identify a large proportion of the total number of peaks detected. However, anything above a 90% mzCloud hit is very likely to be a correct level 2 putative identification and can be used to guide a decision on whether to go forward with a level 1 identification or not. (iv) When faced with limited resources and/or sample size and in the absence of prior knowledge on the metabolites of interest, HILIC chromatography combined with positive ionization is the preferred approach for non-targeted metabolomics due to the number of generated peaks.

## Supporting information

S1 Appendix In-house lipid analysis filter settings & macro-recorder integration settings. (XLSX)

S1 Dataset MzCloud hits with or without standards included in analysis. (XLSX)

S2 Dataset Comparisons between RSD in targeted with non-targeted after different signal drift approaches. (XLSX)

S3 Dataset Comparison of analytes detected and quantified using QqQHILIC and OrbiHILIC+. Post sequence correction with best method (batchCorr) from prestudy for brain & liver and no sequence correction for NIST.
(XLSX)

S4 Dataset Lipid comparison using lipFrag-script to match known lipid fragments with parent ions identified as lipids through a mass-list comparison. RSDs listed for OrbiC18+ and QqQFI analysis respectively.
(XLSX)

S1 Description Compound discoverer workflow settings and example of peak integration. (XLSX)

S1 Table All features potentially analyzed using the two targeted methods (QqQHILIC & QqQFI). (XLSX)

S2 Table Calibration curves and LOD for targeted QqQHILIC method. (XLSX)

S3 Table Sample description and information. (XLSX)

S4 Table Detailed instrument parameters for targeted and non-targeted analyses. (XLSX)

S5 Table Concentrations and RSD of all compounds detected in tissues as well as accuracy in absolute percentage for NIST material. (XLSX)

S6 Table The impact of stationary phase and ionization polarity on the number of features detected during orbitrap analysis (OrbiC18/HILIC +/-). (XLSX)
